# Metagenomic Sequencing Unravels Gene Fragments with Phylogenetic Signatures of O_2_-Tolerant NiFe Membrane-Bound Hydrogenases in Lacustrine Sediment

**DOI:** 10.1007/s00284-015-0846-2

**Published:** 2015-06-05

**Authors:** Jillian M. Couto, Umer Zeeshan Ijaz, Vernon R. Phoenix, Melanie Schirmer, William T. Sloan

**Affiliations:** Division of Infrastructure and Environment, School of Engineering, University of Glasgow, Rankine Building, Level 5, Glasgow, G12 8LT Scotland, UK; School of Geographical and Earth Science, University of Glasgow, Glasgow, G12 8QQ Scotland, UK

## Abstract

Many promising hydrogen technologies utilising hydrogenase enzymes have been slowed by the fact that most hydrogenases are extremely sensitive to O_2_. Within the group 1 membrane-bound NiFe hydrogenase, naturally occurring tolerant enzymes do exist, and O_2_ tolerance has been largely attributed to changes in iron–sulphur clusters coordinated by different numbers of cysteine residues in the enzyme’s small subunit. Indeed, previous work has provided a robust phylogenetic signature of O_2_ tolerance [1], which when combined with new sequencing technologies makes bio prospecting in nature a far more viable endeavour. However, making sense of such a vast diversity is still challenging and could be simplified if known species with O_2_-tolerant enzymes were annotated with information on metabolism and natural environments. Here, we utilised a bioinformatics approach to compare O_2_-tolerant and sensitive membrane-bound NiFe hydrogenases from 177 bacterial species with fully sequenced genomes for differences in their taxonomy, O_2_ requirements, and natural environment. Following this, we interrogated a metagenome from lacustrine surface sediment for novel hydrogenases via high-throughput shotgun DNA sequencing using the Illumina™ MiSeq platform. We found 44 new NiFe group 1 membrane-bound hydrogenase sequence fragments, five of which segregated with the tolerant group on the phylogenetic tree of the enzyme’s small subunit, and four with the large subunit, indicating de novo O_2_-tolerant protein sequences that could help engineer more efficient hydrogenases.

**Electronic supplementary material:**

The online version of this article (doi:10.1007/s00284-015-0846-2) contains supplementary material, which is available to authorized users.

## Introduction

The microbial world is a rich reserve of species and metabolic capabilities, which are being exploited to tackle grand challenges in energy, biotechnology and drug discovery. However, despite our knowledge of this vast diversity, we appear to rely on a few well-characterised organisms in biotechnological applications. Thus, there is a tendency when optimizing a biotechnology process to genetically engineer these organisms rather than seek out more efficient natural organisms. While genetic engineering can be used to enhance performance, it does not always lead to a superior enzyme as has been the case with O_2_ tolerance and NiFe hydrogenases [2].

Hydrogenases are enzymes of great biotechnological interest because they catalyse the H_2_ half-cell reaction [2H^+^ + 2*e′* ⇔ H_2_] that can be manipulated to produce hydrogen from sunlight [3, 4] or sustainably use hydrogen in fuel cells driven by biocatalysts [5, 6]. However, while all three types of hydrogenases, [Ni–Fe], [Fe–Fe] and [Fe-only], can catalyse this reaction for the vast majority of enzymes that we know about, this reaction is severely attenuated, or even irreversibly halted, in the presence of O_2_. Given that O_2_ is either present or produced in every major reaction exploited by these proposed technologies, this intolerance is a major stumbling block that must be overcome [4, 5]. Of all the different types of hydrogenases, the membrane-bound NiFe subtype (MBH) has a few well-characterised O_2_-tolerant members. One in particular, from the bacteria *Ralstonia eutropha*, is currently utilised in experimental enzymatic fuel cells (EFCs) [5]. However, this aero tolerance comes with the cost of both a total bias towards H_2_ oxidation [7–10] and a decreased efficiency [11] when compared with O_2_ sensitive hydrogenases (herein referred to as standard hydrogenases) [12]. As the ideal hydrogenase for many of these technologies would be both oxygen tolerant and efficient, significant work has gone into creating such an enzyme via genetic manipulation [7, 8, 13, 14]. Although efforts thus far have met with little success [2], when combined with research on the structural chemistry [15–21] of MBHs, these studies have delivered significant insight into the mechanisms and gene sequence that are responsible for O_2_ tolerance.

Tolerance has been linked to specific amino acid residues mostly in the small subunit (α) and to a lesser extent in the large subunit (β) of the NiFe MBH. The bulk of the evidence shows that O_2_ tolerance is a function of the proximal Fe–S cluster coordinated by key cysteine residues in the enzyme’s small subunit [1, 8, 14]. Specifically, O_2_-tolerant enzymes possess six cysteine residues (6C group) instead of the typical four conserved cysteine residues (4C group) found in standard hydrogenases. There is also emerging evidence from the enzyme’s large subunit that shows a histidine (H229) residue could serve to further stabilise the proximal cluster in the presence of O_2_ [22, 23]. Taken together, this information produces a reliable phylogenetic signature that can be used to identify potential de novo O_2_-tolerant enzymes from sequence alone.

Indeed, a recent study identified at least 30 additional MBH sequences with the phylogenetic signature suggestive of O_2_ tolerance from publically available fully sequenced genomes of microbes that can be cultured [1]. Cultured isolates represent a small fraction (<1 %) of the diversity of bacteria on the earth [24], so with the vagaries of evolution acting over billions of years on MBH in distantly related organisms it seems reasonable to speculate that there may be an untapped diversity of O_2_-tolerant enzymes in nature. However, sequence information will be of little use if not provided within the context of the organisms’ natural habitat as these organisms have evolved to exploit these diverse environments by fine-tuning their metabolisms to these conditions. Therefore, differences in natural habitats and subtleties of O_2_ metabolisms of an organism could have shaped an enzyme that is more biotechnologically suitable than the ones currently under use.

In this study, we interrogated publically available MBH sequences that had the phylogenetic signature of O_2_ tolerance for differences in taxonomic group, natural environments and oxygen requirements. Following this, we used whole metagenome next generation sequencing to isolate novel O_2_-tolerant NiFe MBH sequence fragments from an environmental sample.

## Methods

### Additions Details can be Found in the Supplementary Text

#### Database Mining: NCBI PSI-Blast Search

NiFe membrane-bound hydrogenase sequences were extracted from the National Centre for Biotechnology Information (www.ncbi.nlm.nih.gov) database that contained 1087 completed microbial genomes at time of query. Searches were conducted with PSI-BLAST [25]. The default setting was changed to return 500 hits. Any query with less than 80 % coverage was eliminated.

#### Environmental Descriptors in SEQenv

The SEQenv pipeline (https://bitbucket.org/seqenv/seqenv/src) retrieves hits to highly similar reference sequences from NCBI and uses a text-mining module to identify a structured and controlled vocabulary of environmental descriptive terms, Environmental Ontology (EnvO) (http://environmentontology.org), mentioned in both associated PubMed abstracts and the “Isolation Source” field entry for the reference hits. We have used version 0.8 of SEQenv that contained a filtered list of approximately 1200 EnvO terms organized into three main branches, namely environmental material, environmental feature and biome. Thus, for each of the 177-hydrogenase sequences, we obtained the EnvO terms along with their frequency of occurrences.

#### Phylogenetic Trees

Trees were constructed with MrBayes v 3.2 [26] using a model that integrated over a set of fixed amino acid matrices (aamodelpr = mixed) [27] with no heated chains. The number of cycles for the MCMC algorithm was set to 2,500,000 generations, with trees sampled every 500 generations using an MCMC analysis.

#### Environmental Samples and Metagenomic Sequencing

Our main field site was in the Lake Torneträsk region (68°21′N, 19°02′E) in Abisko, Sweden. Sampling was conducted from a rowboat using an Eckmann Grab sampler, which collects the top 10-15 cm of sediment. Sediment samples (water depth = 4 m; temp. = 11.5 °C; pH 7.02 surface water temp. = 14.3 °C; air = 17 °C) were immediately sealed in sterile containers and imported back to the United Kingdom (UK) via a permit granted by the UK Plant Health Service and Science and Advice for Scottish Agriculture (SASA). Metagenomic DNA was extracted using the FastDNA™ SPIN kit for soil (MP biomedicals; Santa Ana, CA, USA). Shotgun libraries were constructed and sequenced (paired-end reads) at the Centre for Genomic Research at the University of Liverpool using the Illumina^®^ MiSeq platform. Sequences utilised in this study are provided as supplementary material.

Paired-end reads were filtered and quality trimmed in ‘Sickle’ [28] with the sliding window approach to trim regions when the average base quality dropped below 20. A 10-bp length threshold was used to discard reads that fall below this length after trimming. IDBA-UD [29], an iterative De Bruijn Graph de novo assembler, was used to assemble contigs by iterating from Kmer size of 21–121 and using a pre-correction of reads before assembly. We obtained assembled contigs with a N50 score of 521 with the length of the largest contig being 104,564 bp. The obtained contigs were then run through ‘Prokka’ [30] to obtain annotated Genbank files containing coding sequence regions (CDS) for each contig.

## Results

**Fig. 1:**
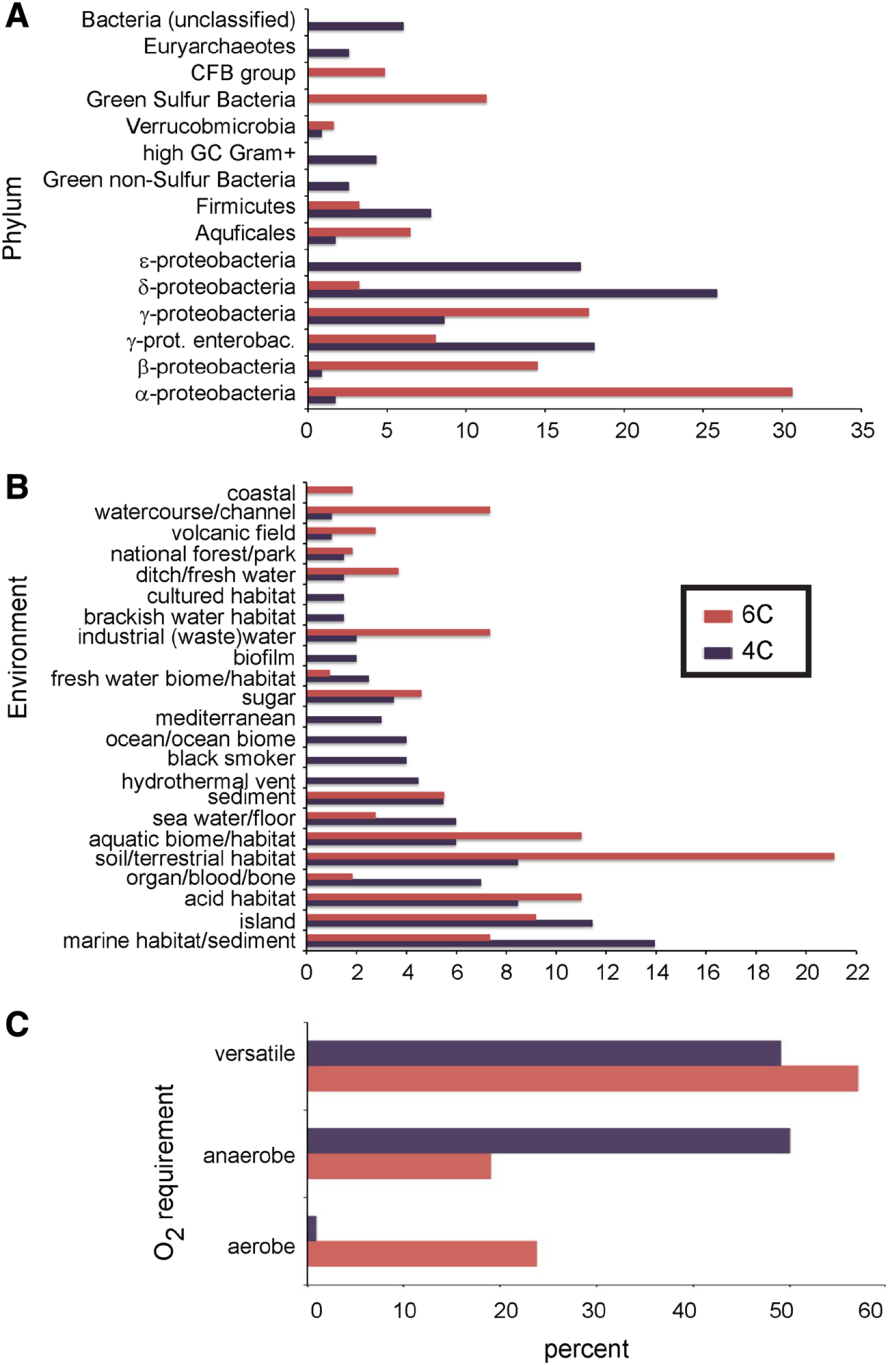
Comparative analysis of Phylum, natural environment and oxygen requirements of 4C and 6C membrane-bound hydrogenases. The distribution of Phyla (**a**), natural environment (**b**) and oxygen requirements (**c**) within the 4C and 6C groups was compared. *Bars* representing the 6C enzymes are shown in *red*, while 4C enzymes are shown in *dark purple*. The horizontal axes show percent per group

Database mining identified 177 sequences for the large and small subunit of the NiFe MBH. We focused on the small subunit, as the evidence for its role in O_2_ tolerance is extremely compelling [1, 8, 14]. Following a sequence alignment to analyse the phylogenetic signature of each enzyme (Table S1), they were classed as O_2_ tolerant if they possessed conserved cysteines (6C; *n* = 63) at six key amino acid positions in the small subunit or standard (4C; *n* = 114) if they had glycine substituted for cysteine at two of these six positions. We then compared the taxonomic group, natural environments and oxygen requirements of these microbes to test for differences. We found an unequal distribution of 4C and 6C enzymes for all three measures (Fig. 1). The taxonomic groups were unequally distributed amongst the 4C and 6C enzymes (Fig. 1a). Notably, the (CFB) group and Green sulphur bacteria (GSB) only contained 6C enzymes, while the Euryarchaeota, Green non-sulphur bacteria (GNS) and ε-proteobacteria only contained 4C enzymes. Similarly, δ-proteobacteria were disproportionately (8.02-fold difference) higher in the 4C group, while the α- (17.77-fold) and β- proteobacteria (16.84-fold) were over-represented in the 6C group.

Natural environments returned from SEQenv were also unequally distributed amongst the two groups of enzymes (Fig. 1b). Overall, the 4C group contained organisms that inhabited more saline, marine environments and environments of geothermal significance, while organisms with 6C enzymes appeared to be more prevalent in terrestrial and freshwater environments.

Microbes were classed as aerobes, anaerobes or versatile in order to assess their O_2_ requirements. The versatile organisms appeared in roughly equal proportions in both the 4C and 6C groups, while there was a striking difference between the distribution of aerobes and anaerobes (Fig. 1c). The 6C group contained 28.5-fold more aerobes than the 4C group. Conversely, there were 2.54-fold more anaerobes in the 4C group compared with the 6C group.

Aquatic ecosystems are important for the flux of H_2_ in and out of the environment and have been investigated previously for new hydrogenases [31, 32]. To explore this diversity further, we extracted metagenomic DNA from sediment from a subarctic lake in North Sweden (68°21′N, 19°02′E) and directly sequenced the metagenome using the Illumina™ MiSeq platform. Following contig assembly, 241 sequences could be annotated with the enzyme commission (E.C.) number 1.12. -. -, identifying them as hydrogenases. Of these, 18 sequences could be identified as group 1 MBH small subunits, and 26 sequences could be identified as the large subunit of the group 1 MBH. These sequences ranged in length from full proteins to 35 amino acid fragments. All 44 sequences were compared against the NCBI non-redundant protein sequence database using PSI-BLAST [25]. Of the small subunit sequences, one 362 amino acid query (sequence 20) was an exact match to a known sequence representing multiple species from the *Rhodocyclaceae* (GI: 518758527) family (Table S2). For the large subunit, a 38 amino acid fragment was an exact match to *Asticcacaulis* sp. *AC466* (GI: 557832447). The rest were partial matches, suggesting that they could be sequences from previously uncharacterised organisms (Table S2).

**Fig. 2:**
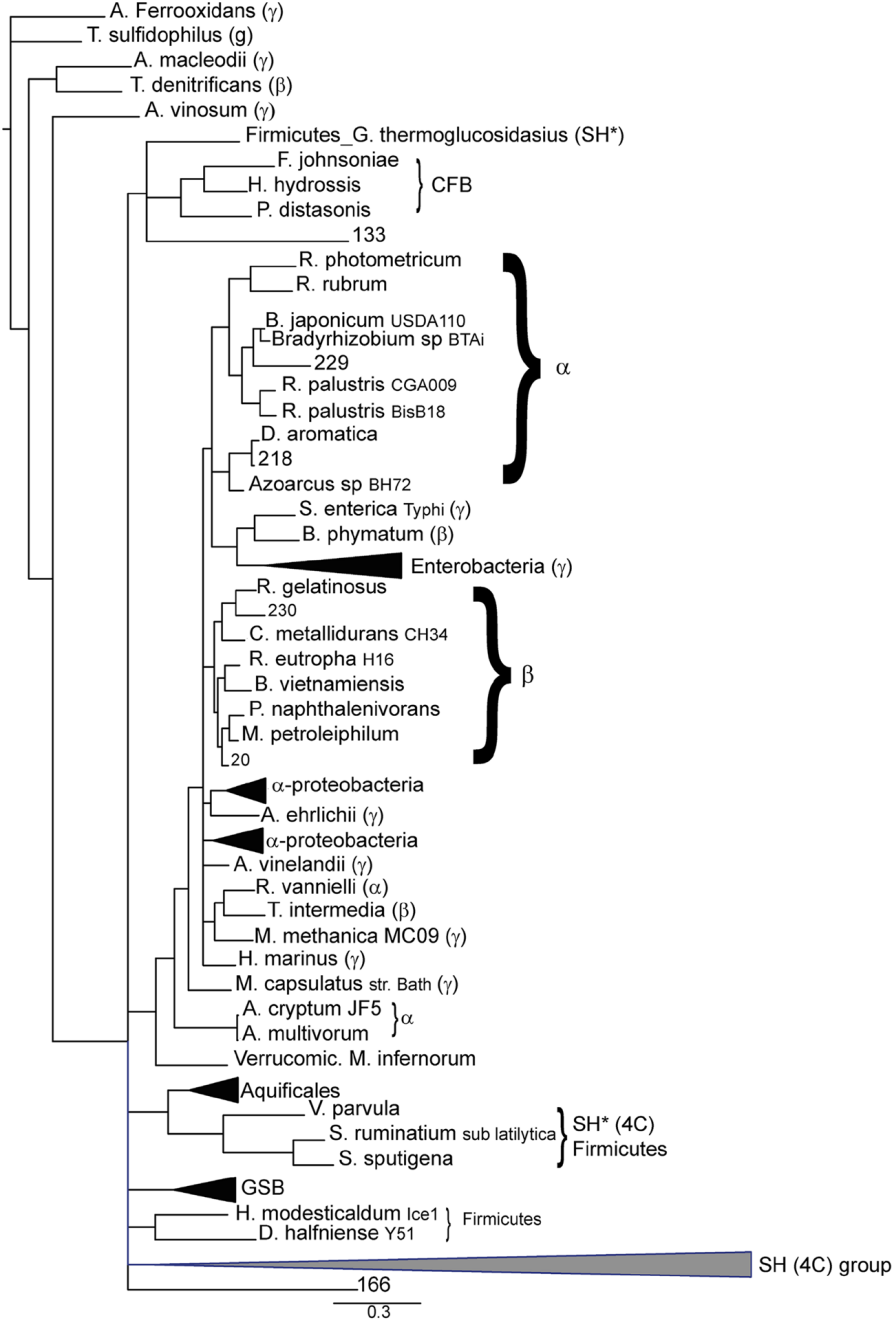
Phylogenetic tree of sequences for the enzyme’s small subunit with segregating 6C environmental metagenomic fragments. The fragments 133, 229, 218, 230 and 20 segregate within the 6C group. The 4C group (*grey triangle*) has been collapsed but can be viewed in detail in Figure S4. Fragment 166 appears to form a distinct group. The scale refers to 0.3 expected changes per site. An *asterisk* marks the four standard hydrogenase (SH)/4C hydrogenases from the *Firmicutes* phylum that cluster within the 6C group. Unless otherwise indicated, all enzymes are 6C

Utilising the 177 database sequences and a Bayesian inference, we estimated the phylogeny of both the small and large subunit with a Markov Chain Monte Carlo (MCMC) approximation to construct trees (Figures S1–S5) onto which we could place the 44 sequence fragments from our metagenomic analysis. For the small subunit, all 18 sequences appeared on different branches with distinct lengths (Fig. 2, 6C; Figure S4, 4C). Of these, sequences 133, 229, 218, 230 and 20 segregated within the 6C group (Fig. 2). Sequence 20 in particular is a full-length protein and has the critical cysteine residues, α_62_ and α_163_. Sequence 218 is a partial fragment that also contains a cysteine residue at α_163_. The Fe–S cluster co-ordinating region was not recovered for the rest of the sequences; however, both sequence 229 and 230 segregate with the 6C enzymes suggesting that they too are O_2_ tolerant. Fragment 133 was only 55 residues in length and grouped with both the 6C CFB organisms and the 4C *G. thermoglucosidasius*, suggesting that it could be from either group.

Similarly, the 26 large subunit sequences appeared to be distinct species, with fragment numbers 6, 39, 232 and 181 segregating with the 6C enzymes (Figure S5). Of these, sequence 232 is nearly full-length and segregates with *R. gelatinosus* and *C. metallidurans*, two well-characterised O_2_-tolerant hydrogenases, suggesting that it too is an O_2_-tolerant hydrogenase from the *β*-*proteobacteria* phylum. The rest were short fragments but grouped with 6C containing hydrogenases.

## Discussion

The immediate need for efficient biotechnologies to solve current problems such as sustainable sources of energy has driven us to explore the microbial landscape for unique organisms and enzymes. However on its own, sequence information is not enough and needs to be paired with contextual information about the organism and the environment it inhabits. The observation that the 4C and 6C enzymes are not randomly distributed across phyla, natural environments or oxygen requirements suggests that these factors have influenced the evolution of these enzymes. Overall, there appears to be a shift from harsh, nutrient poor environments, to more anodyne, nutrient-rich environment. The 4C enzyme grouping mostly contained organisms that could metabolise sulphur and metals anaerobically. The 6C enzyme group contained organisms with aerobic metabolisms as well as many species that could either photosynthesize or metabolise nitrogen. This supports the idea of a redox up-shift via high-potential terminal electron acceptors within the 6C group [1]. An exception is the enzyme from *R. eutropha*, utilised in some experimental biotechnologies [3–5]. This organism exploits the relatively redox poor “*Knallgas*” reaction despite being part of the 6C group. A more superior enzyme could be purified from phylogenetically related organisms that exploit high-potential redox reactions. In particular, the purple non-sulphur bacteria display an array of metabolic capabilities and are actively being investigated for their potential in H_2_ technologies [33–40]. Having evolved different modifications, some of which may provide increased efficiency, exploring these groups might greatly benefit the engineered systems.

The presence of O_2_ in the organism’s natural environment could also affect enzyme efficiency. In environments with a flux of O_2_, there is still the possibility that when O_2_ is present, the MBH is either not expressed or is expressed but has a lowered efficiency. A relationship between expression, aerobicity and enzyme function was demonstrated with hyd-5 in *S. enterica* [22, 41]. Twenty-four percent of the enzymes from our study were detected in predominantly aerobic environments with a potentially constant exposure to O_2_. Therefore, one could speculate that aerobes would possess hydrogenases that are extremely tolerant and potentially more efficient than the ones in the versatile group and the obligate anaerobes. Indeed, in experiments, the aerobe *H. marinus* retained a higher percentage of activity compared to the microaerophilic *R. eutropha* after exposure to air [42]. The aerobes *B. vietnamiensisG4*, *P. naphthalenivorans CJ2* and *M. petroleiphilum PM1* have phylogenetically related hydrogenases to *R. eutropha* (Figure S1) that might have evolved greater efficiency due to their aerobic heterotrophic lifestyles.

The current study recovered MBH sequence fragments via shotgun high-throughput sequencing of metagenomic DNA from an environmental sample. Although the number of MBH sequence fragments recovered in our study was lower than expected, it is comparable to work utilising similar techniques to discover novel hydrogenases from the global ocean survey of surface waters [32]. Of the five new 6C sequences, four segregate with aerobic/facultative heterotrophic α- and β- proteobacteria on the small subunit tree, and similar to their cultured neighbours, could also make use of high-potential redox couples. The full-length small subunit sequence 20 from the *Rhodocyclales* family (Table S2) segregates with the aerobic β-proteobacteria *Methylibium petroleiphilum* and *Polaromonas naphthalenivorans*. Both are aquatic aerobic heterotrophs that can use methyl tert-butyl ether [43] and Naphthalene [44], respectively, as sole carbon sources. Similarly, the close-to-full-length fragment 218 segregates with *Decloromonas aromatica* species and is most likely from this or a closely related organism.

The phylogenetic tree and analyses of taxonomic groups, natural environments and oxygen requirements enabled us to place all 44 de novo MBH sequence fragment amongst the 177 MBH identified from the database search providing a powerful tool for bio-inspired enzyme engineering. Using the database analysis, we can now make use of well-characterised biology and couple it to the uniqueness offered by an untapped reserve of natural diversity, greatly enhancing a simple BLAST search. It stands to reason that these de novo sequences contain novel combinations of amino acid residues that could be utilised to engineer the hydrogenases from organisms that lend themselves well to pure-culture. In addition, identifying related sequences can target primer design and identify genomes of organisms that can serve as a scaffold for downstream procedures to assemble and retrieve ‘missing’ gene information in order to re-create a full enzyme.

## Electronic Supplementary Material

Supplementary material 1 (DOCX 3630 kb)
