# Supplementary material for: Metagenomic Sequencing Unravels Gene Fragments with Phylogenetic Signatures of O2-Tolerant NiFe Membrane-Bound Hydrogenases in Lacustrine Sediment
Source: Curr Microbiol. 2015 Jun 5;71(2):296–302. doi: 10.1007/s00284-015-0846-2 (PMC4486115; doi:10.1007/s00284-015-0846-2)
Supplement: Supplementary file 1 — Supplementary material 1 (DOCX 3630 kb) [file 284_2015_846_MOESM1_ESM.docx]

**Supplementary Information for**

**Metagenomic sequencing unravels gene fragments with phylogenetic signatures of O_2_ tolerant NiFe membrane bound hydrogenases in lacustrine sediment**

Jillian M. Couto^1*^, Umer Zeeshan Ijaz^1^, Vernon R. Phoenix^2^, Melanie Schirmer^1^, William T. Sloan^1^

1: Division of Infrastructure and Environment, School of Engineering; 2: School of Geographical and Earth Science, University of Glasgow, Glasgow G12 8QQ

*Corresponding Author:

Dr. Jillian M. Couto

University of Glasgow

School of Engineering, Infrastructure and Environment

Rankine Building, Level 5

Glasgow, G12 8LT

[Jillian.Couto@glasgow.ac.uk](mailto:Jillian.Couto@glasgow.ac.uk)

Ph: +44(0) 141 330 6311

Fax: +44(0) 141 330 4907

Working title: oxygen tolerant hydrogenases from lacustrine sediment

**Supplementary Methods**

**NCBI PSI-Blast search**

Searches were conducted with PSI-BLAST [1], using the amino acid sequence for the small subunit of the MBH from *R. eutropha* H16 (GI: 400231) as a query for tolerant enzymes and *D. fructosovorans JJ* (GI: 205831249) as the query for standard enzymes. The default setting was changed to return 500 hits. Any query with less than 80% coverage was eliminated. As were any hits with obvious gene names that were not NiFe hydrogenases and duplicates, sub-strains, and serovars. There was an almost complete overlap between the two searches. To further filter the lists into tolerant and standard hydrogenases, all hits were aligned with Clustal Omega (www.ebi.ac.uk/Tools/msa/clustalo) and hits containing the cysteine residues at positions 62 and 163 of the small (α) subunit (Table S1) were classed as “tolerant” and hits containing glycine residues at those positions were classed as “standard”. Residues at key positions in both subunits are outlined in Table S1. In addition all retained hits contained the RRxFxK twin Arginine motif characteristic of the signal peptide in the small subunit [2]. Although not essential for O_2_ tolerance, the RRxFxK motif was utilised as it is critical for functioning of all group 1 membrane bound NiFe hydrogenases and focussing on critical residues is one way of reducing the likelihood of isolating pseudogenes from a database [2].

**Phylogenetic trees**

For the MrBayes analysis, the first 25% of runs were discarded as the burn-in phase. All trees analysed appeared to support the WAG [3] model with both a minimum and maximum probability = 1.000. The consensus phylogenetic trees were presented in the current study. Statistical certainty was assessed using the potential scale reduction factor, which approached 1.000 as parallel MCMC runs converged, the average standard deviation between runs, and the branch probabilities for each tree.

**Next-generation sequence processing and Bioinformatics**

The IDBA-UD [4] assembler was utilised as it can incorporate uneven sequencing depth and uses progressive depth on contigs to reduce errors in high-depth regions.

We obtained assembled contigs with a N50 score of 521 with the length of the largest contig being 104,564 bp. The obtained contigs were then run through ‘Prokka’ [5] to obtain annotated Genbank files. A total of 252,176 proteins were discovered among which 152,933 were "hypothetical proteins" i.e. large open reading frames that do not have a characterised homologue in the NCBI protein database. We then identified 241 hydrogenase sequences using the enzyme commission (EC) number 1.12.x.x. Further alignments revealed that 18 of these could be identified as group 1 MBH small subunits and 26 sequences could be identified as the large subunit of the group 1 MBH (Table S2). These sequence fragments ranged in length from full proteins to 35 amino acid fragments.

| TAXA ID | Organism | Phylum | O_2_ sens. | α_60_ | α_62_ | α_63_ | α_158_ | α_163_ | α_192_ | β_73_ | β_229_ |
| --- | --- | --- | --- | --- | --- | --- | --- | --- | --- | --- | --- |
| 258594 | Rhodopseudomonas palustris CGA009 | α-proteo. | 6C | C | C | C | C | C | C | Q | H |
| 269796 | Rhodospirillum rubrum ATCC 11170 | α-proteo. | 6C | C | C | C | C | C | C | Q | H |
| 648757 | Rhodomicrobium vannielii ATCC 17100 | α-proteo. | 6C | C | C | C | C | C | C | Q | H |
| 1150469 | Rhodospirillum photometricum DSM 122 | α-proteo. | 6C | C | C | C | C | C | C | Q | H |
| 224324 | Aquifex aeolicus VF5 | aquificales | 6C | C | C | C | C | C | C | Q | H |
| 380749 | Hydrogenobaculum sp. Y04AAS1 species | aquificales | 6C | C | C | C | C | C | C | Q | H |
| 608538 | Hydrogenobacter thermophilus TK-6 | aquificales | 6C | C | C | C | C | C | C | Q | H |
| 638303 | Thermocrinis albus DSM 14484 | aquificales | 6C | C | C | C | C | C | C | Q | H |
| 376686 | Flavobacterium johnsoniae UW101 | CFB grp | 6C | C | C | C | C | C | C | Q | H |
| 138119 | Desulfitobacterium hafniense Y51 | firmicutes | 6C | C | C | C | C | C | C | Q | H |
| 498761 | Heliobacterium modesticaldum Ice1 | firmicutes | 6C | C | C | C | C | C | C | Q | H |
| 187272 | Alkalilimnicola ehrlichii MLHE-1 | γ-proteo. | 6C | C | C | C | C | C | C | Q | H |
| 857087 | Methylomonas methanica MC09 | γ-proteo. | 6C | C | C | C | C | C | C | Q | H |
| 481448 | Methylacidiphilum infernorum V4 | verrucomic. | 6C | C | C | C | C | C | C | Q | H |
| 435591 | Parabacteroides distasonis ATCC 8503 | CFB grp | 6C | C | C | C | C | C | C | G | H |
| 760192 | Haliscomenobacter hydrossis DSM 1100 | CFB grp | 6C | C | C | C | C | C | C | G | H |
| 78245 | Xanthobacter autotrophicus Py2 | α-proteo. | 6C | C | C | C | C | C | C | E | H |
| 137722 | Azospirillum sp. B510 species | α-proteo. | 6C | C | C | C | C | C | C | E | H |
| 224911 | Bradyrhizobium japonicum USDA 110 | α-proteo. | 6C | C | C | C | C | C | C | E | H |
| 272942 | Rhodobacter capsulatus SB 1003 | α-proteo. | 6C | C | C | C | C | C | C | E | H |
| 272943 | Rhodobacter sphaeroides 2.4.1 | α-proteo. | 6C | C | C | C | C | C | C | E | H |
| 288000 | Bradyrhizobium sp. BTAi1 species | α-proteo. | 6C | C | C | C | C | C | C | E | H |
| 316056 | Rhodopseudomonas palustris BisB18 | α-proteo. | 6C | C | C | C | C | C | C | E | H |
| 318586 | Paracoccus denitrificans PD1222 | α-proteo. | 6C | C | C | C | C | C | C | E | H |
| 349102 | Rhodobacter sphaeroides ATCC 17025 | α-proteo. | 6C | C | C | C | C | C | C | E | H |
| 349163 | Acidiphilium cryptum JF-5 | α-proteo. | 6C | C | C | C | C | C | C | E | H |
| 395963 | Beijerinckia indica subsp. indica ATCC 9039 | α-proteo. | 6C | C | C | C | C | C | C | E | H |
| 438753 | Azorhizobium caulinodans ORS 571 | α-proteo. | 6C | C | C | C | C | C | C | E | H |
| 504832 | Oligotropha carboxidovorans OM5 | α-proteo. | 6C | C | C | C | C | C | C | E | H |
| 717785 | Hyphomicrobium sp. MC1 species | α-proteo. | 6C | C | C | C | C | C | C | E | H |
| 926570 | Acidiphilium multivorum AIU301 | α-proteo. | 6C | C | C | C | C | C | C | E | H |
| 62928 | Azoarcus sp. BH72 species | β-proteo. | 6C | C | C | C | C | C | C | E | H |
| 75379 | Thiomonas intermedia K12 | β-proteo. | 6C | C | C | C | C | C | C | E | H |
| 159087 | Dechloromonas aromatica RCB | β-proteo. | 6C | C | C | C | C | C | C | E | H |
| 266264 | Cupriavidus metallidurans CH34 | β-proteo. | 6C | C | C | C | C | C | C | E | H |
| 269482 | Burkholderia vietnamiensis G4 | β-proteo. | 6C | C | C | C | C | C | C | E | H |
| 365044 | Polaromonas naphthalenivorans CJ2 | β-proteo. | 6C | C | C | C | C | C | C | E | H |
| 381666 | Ralstonia eutropha H16 | β-proteo. | 6C | C | C | C | C | C | C | E | H |
| 391038 | Burkholderia phymatum STM815 | β-proteo. | 6C | C | C | C | C | C | C | E | H |
| 420662 | Methylibium petroleiphilum PM1 | β-proteo. | 6C | C | C | C | C | C | C | E | H |
| 363253 | Lawsonia intracellularis PHE/MN1-00 | δ-proteo. | 6C | C | C | C | C | C | C | E | H |
| 525146 | Desulfovibrio desulfuricans str. ATCC 27774 | δ-proteo. | 6C | C | C | C | C | C | C | E | H |
| 99287 | Salmonella enterica Typhimurium str. LT2 | enterobac. | 6C | C | C | C | C | C | C | E | H |
| 198214 | Shigella flexneri 2a str. 301 | enterobac. | 6C | C | C | C | C | C | C | E | H |
| 300269 | Shigella sonnei Ss046 | enterobac. | 6C | C | C | C | C | C | C | E | H |
| 331111 | Escherichia coli E24377A | enterobac. | 6C | C | C | C | C | C | C | E | H |
| 358708 | Shigella dysenteriae 1012 | enterobac. | 6C | C | C | C | C | C | C | E | H |
| 439847 | Salmonella enterica Saintpaul str. SARA29 | enterobac. | 6C | C | C | C | C | C | C | E | H |
| 498217 | Edwardsiella tarda EIB202 | enterobac. | 6C | C | C | C | C | C | C | E | H |
| 500638 | Edwardsiella tarda ATCC 23685 | enterobac. | 6C | C | C | C | C | C | C | E | H |
| 585054 | Escherichia fergusonii ATCC 35469 | enterobac. | 6C | C | C | C | C | C | C | E | H |
| 634503 | Edwardsiella ictaluri 93-146 | enterobac. | 6C | C | C | C | C | C | C | E | H |
| 766141 | Shigella boydii 5216-82 | enterobac. | 6C | C | C | C | C | C | C | E | H |
| 243233 | Methylococcus capsulatus str. Bath | γ-proteo. | 6C | C | C | C | C | C | C | E | H |
| 322710 | Azotobacter vinelandii DJ | γ-proteo. | 6C | C | C | C | C | C | C | E | H |
| 1123513 | Hydrogenovibrio marinus DSM 11271 | γ-proteo. | 6C | C | C | C | C | C | C | E | H |
| 1096 | Chlorobium phaeobacteroides species | GSB | 6C | C | C | C | C | C | C | E | H |
| 290315 | Chlorobium limicola DSM 245 | GSB | 6C | C | C | C | C | C | C | E | H |
| 290512 | Prosthecochloris aestuarii DSM 271 | GSB | 6C | C | C | C | C | C | C | E | H |
| 319225 | Chlorobium luteolum DSM 273 | GSB | 6C | C | C | C | C | C | C | E | H |
| 324925 | Pelodictyon phaeoclathratiforme BU-1 | GSB | 6C | C | C | C | C | C | C | E | H |
| 517417 | Chlorobaculum parvum NCIB 8327 | GSB | 6C | C | C | C | C | C | C | E | H |
| 517418 | Chloroherpeton thalassium ATCC 35110 | GSB | 6C | C | C | C | C | C | C | E | H |
| 552811 | Dehalogenimonas lykanthroporepellens BL-DC-9 | GNS bacteria | 4C | C | G | C | C | G | C | S | H |
| 234267 | Candidatus Solibacter usitatus Ellin6076 | bacteria | 4C | C | G | C | C | G | C | Q | N |
| 795359 | Thermodesulfobacterium sp. OPB45 | bacteria | 4C | C | G | C | C | G | C | Q | N |
| 290397 | Anaeromyxobacter dehalogenans 2CP-C | δ-proteo. | 4C | C | G | C | C | G | C | Q | N |
| 404589 | Anaeromyxobacter sp. Fw109-5 | δ-proteo. | 4C | C | G | C | C | G | C | Q | N |
| 447217 | Anaeromyxobacter sp. K | δ-proteo. | 4C | C | G | C | C | G | C | Q | N |
| 156889 | Magnetococcus marinus MC-1 | α-proteo. | 4C | C | G | P | C | G | C | Q | H |
| 342108 | Magnetospirillum magneticum AMB-1 | α-proteo. | 4C | C | G | C | C | G | C | Q | H |
| 292415 | Thiobacillus denitrificans ATCC 25259 | β-proteo. | 4C | C | G | C | C | G | C | Q | H |
| 289376 | Thermodesulfovibrio yellowstonii DSM 11347 | bacteria | 4C | C | G | C | C | G | C | Q | H |
| 639282 | Deferribacter desulfuricans SSM1 | bacteria | 4C | C | G | D | C | G | C | Q | H |
| 667014 | Thermodesulfatator indicus DSM 15286 | bacteria | 4C | C | G | C | C | G | C | Q | H |
| 717231 | Flexistipes sinusarabici DSM 4947 | bacteria | 4C | C | G | D | C | G | C | Q | H |
| 768670 | Calditerrivibrio nitroreducens DSM 19672 | bacteria | 4C | C | G | D | C | G | C | Q | H |
| 882 | Desulfovibrio vulgaris str. Hildenborough | δ-proteo. | 4C | C | G | C | C | G | C | Q | H |
| 883 | Desulfovibrio vulgaris str. 'Miyazaki F' | δ-proteo. | 4C | C | G | C | C | G | C | Q | H |
| 177437 | Desulfobacterium autotrophicum HRM2 | δ-proteo. | 4C | C | G | C | C | G | C | Q | H |
| 177439 | Desulfotalea psychrophila LSv54 | δ-proteo. | 4C | C | G | C | C | G | C | Q | H |
| 207559 | Desulfovibrio alaskensis G20 | δ-proteo. | 4C | C | G | C | C | G | C | Q | H |
| 243231 | Geobacter sulfurreducens PCA | δ-proteo. | 4C | C | G | C | C | G | C | Q | H |
| 269799 | Geobacter metallireducens GS-15 | δ-proteo. | 4C | C | G | D | C | G | C | Q | H |
| 335543 | Syntrophobacter fumaroxidans MPOB | δ-proteo. | 4C | C | G | C | C | G | C | Q | H |
| 351605 | Geobacter uraniireducens Rf4 | δ-proteo. | 4C | C | G | D | C | G | C | Q | H |
| 398767 | Geobacter lovleyi SZ | δ-proteo. | 4C | C | G | D | C | G | C | Q | H |
| 404380 | Geobacter bemidjiensis Bem | δ-proteo. | 4C | C | G | C | C | G | C | Q | H |
| 439235 | Desulfatibacillum alkenivorans AK-01 | δ-proteo. | 4C | C | G | C | C | G | C | Q | H |
| 443143 | Geobacter sp. M18 | δ-proteo. | 4C | C | G | D | C | G | C | Q | H |
| 443144 | Geobacter sp. M21 | δ-proteo. | 4C | C | G | C | C | G | C | Q | H |
| 485915 | Desulfohalobium retbaense DSM 5692 | δ-proteo. | 4C | C | G | C | C | G | C | Q | H |
| 525897 | Desulfomicrobium baculatum DSM 4028 | δ-proteo. | 4C | C | G | C | C | G | C | Q | H |
| 526222 | Desulfovibrio salexigens DSM 2638 | δ-proteo. | 4C | C | G | C | C | G | C | Q | H |
| 573370 | Desulfovibrio magneticus RS-1 | δ-proteo. | 4C | C | G | C | C | G | C | Q | H |
| 577650 | Desulfobulbus propionicus DSM 2032 | δ-proteo. | 4C | C | G | C | C | G | C | Q | H |
| 589865 | Desulfurivibrio alkaliphilus AHT2 | δ-proteo. | 4C | C | G | C | C | G | C | Q | H |
| 596151 | Desufovibrio fructosovorans JJ | δ-proteo. | 4C | C | G | C | C | G | C | Q | H |
| 643562 | Desulfovibrio aespoeensis Aspo-2 | δ-proteo. | 4C | C | G | C | C | G | C | Q | H |
| 644282 | Desulfarculus baarsii DSM 2075 | δ-proteo. | 4C | C | G | C | C | G | C | Q | H |
| 690850 | Desulfovibrio africanus str. Walvis Bay | δ-proteo. | 4C | C | G | C | C | G | C | Q | H |
| 706587 | Desulfomonile tiedjei DSM 6799 | δ-proteo. | 4C | C | G | C | C | G | C | Q | H |
| 760142 | Hippea maritima DSM 10411 | δ-proteo. | 4C | C | G | C | C | G | C | Q | H |
| 1121448 | Desulfovibrio gigas DSM1382 | δ-proteo. | 4C | C | G | C | C | G | C | Q | H |
| 235279 | Helicobacter hepaticus ATCC 51449 | ε-proteo. | 4C | C | G | C | C | G | C | Q | H |
| 273121 | Wolinella succinogenes DSM 1740 | ε-proteo. | 4C | C | G | C | C | G | C | Q | H |
| 306263 | Campylobacter lari RM2100 | ε-proteo. | 4C | C | G | C | C | G | C | Q | H |
| 360104 | Campylobacter concisus 13826 | ε-proteo. | 4C | C | G | C | C | G | C | Q | H |
| 360106 | Campylobacter fetus subsp. fetus 82-40 | ε-proteo. | 4C | C | G | C | C | G | C | Q | H |
| 360107 | Campylobacter hominis ATCC BAA-381 | ε-proteo. | 4C | C | G | C | C | G | C | Q | H |
| 367737 | Arcobacter butzleri RM4018 | ε-proteo. | 4C | C | G | C | C | G | C | Q | H |
| 525898 | Sulfurospirillum deleyianum DSM 6946 | ε-proteo. | 4C | C | G | C | C | G | C | Q | H |
| 572480 | Arcobacter nitrofigilis DSM 7299 | ε-proteo. | 4C | C | G | C | C | G | C | Q | H |
| 598659 | Nautilia profundicola AmH | ε-proteo. | 4C | C | G | C | C | G | C | Q | H |
| 679897 | Helicobacter mustelae 12198 | ε-proteo. | 4C | C | G | C | C | G | C | Q | H |
| 760154 | Sulfurospirillum barnesii SES-3 | ε-proteo. | 4C | C | G | C | C | G | C | Q | H |
| 936155 | Helicobacter felis ATCC 49179 | ε-proteo. | 4C | C | G | C | C | G | C | Q | H |
| 1002804 | Helicobacter bizzozeronii CIII-1 | ε-proteo. | 4C | C | G | C | C | G | C | Q | H |
| 1172562 | Helicobacter cinaedi PAGU611 | ε-proteo. | 4C | C | G | C | C | G | C | Q | H |
| 198628 | Dickeya dadantii 3937 | enterobac. | 4C | C | G | C | C | G | C | Q | H |
| 218491 | Pectobacterium atrosepticum SCRI1043 | enterobac. | 4C | C | G | C | C | G | C | Q | H |
| 218493 | Salmonella bongori NCTC 12419 | enterobac. | 4C | C | G | C | C | G | C | Q | H |
| 290338 | Citrobacter koseri ATCC BAA-895 | enterobac. | 4C | C | G | C | C | G | C | Q | H |
| 393305 | Yersinia enterocolitica subsp. enterocolitica 8081 | enterobac. | 4C | C | G | C | C | G | C | Q | H |
| 529507 | Proteus mirabilis HI4320 | enterobac. | 4C | C | G | C | C | G | C | Q | H |
| 561229 | Dickeya zeae Ech1591 | enterobac. | 4C | C | G | C | C | G | C | Q | H |
| 630626 | Escherichia blattae DSM 4481 | enterobac. | 4C | C | G | C | C | G | C | Q | H |
| 637910 | Citrobacter rodentium ICC168 | enterobac. | 4C | C | G | C | C | G | C | Q | H |
| 701347 | Enterobacter cloacae SCF1 | enterobac. | 4C | C | G | C | C | G | C | Q | H |
| 572546 | Archaeoglobus profundus DSM 5631 | enterobac. | 4C | C | G | C | C | G | C | Q | H |
| 589924 | Ferroglobus placidus DSM 10642 | euryarch. | 4C | C | G | C | C | G | C | Q | H |
| 693661 | Archaeoglobus veneficus SNP6 | euryarch. | 4C | C | G | C | C | G | C | Q | H |
| 246194 | Carboxydothermus hydrogenoformans Z-2901 | firmicutes | 4C | C | G | C | C | G | C | Q | H |
| 370438 | Pelotomaculum thermopropionicum SI | firmicutes | 4C | C | G | C | C | G | C | Q | H |
| 546271 | Selenomonas sputigena ATCC 35185 | firmicutes | 4C | C | G | C | C | G | C | Q | H |
| 634956 | Geobacillus thermoglucosidasius C56-YS93 | firmicutes | 4C | C | G | C | C | G | C | Q | H |
| 686660 | Veillonella parvula ATCC 17745 | firmicutes | 4C | C | G | C | C | G | C | Q | H |
| 927704 | Selenomonas ruminantium sub. lactilytica TAM6421 | firmicutes | 4C | C | G | C | C | G | C | Q | H |
| 1051632 | Sulfobacillus acidophilus TPY | firmicutes | 4C | C | G | N | C | G | C | Q | H |
| 729 | Haemophilus parainfluenzae | γ-proteo. | 4C | C | G | C | C | G | C | Q | H |
| 211586 | Shewanella oneidensis MR-1 | γ-proteo. | 4C | C | G | C | C | G | C | Q | H |
| 221988 | Mannheimia succiniciproducens MBEL55E | γ-proteo. | 4C | C | G | C | C | G | C | Q | H |
| 225849 | Shewanella piezotolerans WP3 | γ-proteo. | 4C | C | G | C | C | G | C | Q | H |
| 318167 | Shewanella frigidimarina NCIMB 400 | γ-proteo. | 4C | C | G | C | C | G | C | Q | H |
| 323850 | Shewanella loihica PV-4 | γ-proteo. | 4C | C | G | C | C | G | C | Q | H |
| 325240 | Shewanella baltica OS155 | γ-proteo. | 4C | C | G | C | C | G | C | Q | H |
| 339671 | Actinobacillus succinogenes 130Z | γ-proteo. | 4C | C | G | C | C | G | C | Q | H |
| 380394 | Acidithiobacillus ferrooxidans ATCC 53993 | γ-proteo. | 4C | C | G | C | C | G | C | Q | H |
| 380703 | Aeromonas hydrophila sub. hydrophila ATCC 7966 | γ-proteo. | 4C | C | G | C | C | G | C | Q | H |
| 382245 | Aeromonas salmonicida sub. salmonicida A449 | γ-proteo. | 4C | C | G | C | C | G | C | Q | H |
| 396588 | Thioalkalivibrio sulfidophilus HL-EbGr7 | γ-proteo. | 4C | C | G | C | C | G | C | Q | H |
| 398579 | Shewanella pealeana ATCC 700345 | γ-proteo. | 4C | C | G | C | C | G | C | Q | H |
| 458817 | Shewanella halifaxensis HAW-EB4 | γ-proteo. | 4C | C | G | C | C | G | C | Q | H |
| 550540 | Ferrimonas balearica DSM 9799 | γ-proteo. | 4C | C | G | C | C | G | C | Q | H |
| 572477 | Allochromatium vinosum DSM 180 | γ-proteo. | 4C | C | G | C | C | G | C | Q | H |
| 634176 | Aggregatibacter aphrophilus NJ8700 | γ-proteo. | 4C | C | G | C | C | G | C | Q | H |
| 754261 | Actinobacillus pleuropneumoniae serovar 12 str. 1096 | γ-proteo. | 4C | C | G | C | C | G | C | Q | H |
| 907488 | Aggregatibac. actinomycetemcomitans str. SC1083 | γ-proteo. | 4C | C | G | C | C | G | C | Q | H |
| 998088 | Aeromonas veronii B565 | γ-proteo. | 4C | C | G | C | C | G | C | Q | H |
| 1004786 | Alteromonas macleodii AltDE1 | γ-proteo. | 4C | C | G | C | C | G | C | Q | H |
| 357808 | Roseiflexus sp. RS-1 | GNS | 4C | C | G | N | C | G | C | Q | H |
| 383372 | Roseiflexus castenholzii DSM 13941 | GNS | 4C | C | G | N | C | G | C | Q | H |
| 298653 | Frankia sp. EAN1pec | high GC Gr+ | 4C | C | G | D | C | G | C | Q | H |
| 469378 | Cryptobacterium curtum DSM 15641 | high GC Gr+ | 4C | C | G | C | C | G | C | Q | H |
| 471855 | Slackia heliotrinireducens DSM 20476 | high GC Gr+ | 4C | C | G | C | C | G | C | Q | H |
| 548479 | Mobiluncus curtisii ATCC 43063 | high GC Gr+ | 4C | C | G | C | C | G | C | Q | H |
| 123214 | Persephonella marina EX-H1 | aquificales | 4C | C | G | N | C | G | C | M | H |
| 204536 | Sulfurihydrogenibium azorense Az-Fu1 | aquificales | 4C | C | G | N | C | G | C | M | H |
| 387092 | Nitratiruptor sp. SB155-2 | ε-proteo. | 4C | C | G | N | C | G | C | M | H |
| 387093 | Sulfurovum sp. NBC37-1 | ε-proteo. | 4C | C | G | N | C | G | C | M | H |
| 709032 | Sulfuricurvum kujiense DSM 16994 | ε-proteo. | 4C | C | G | N | C | G | C | M | H |
| 749222 | Nitratifractor salsuginis DSM 16511 | ε-proteo. | 4C | C | G | N | C | G | C | M | H |
| 944547 | Arcobacter sp. L | ε-proteo. | 4C | C | G | N | C | G | C | M | H |
| 698966 | Corynebacterium diphtheriae 241 | high GC Gr+ | 4C | C | G | C | C | G | C | G | H |
| 635013 | Thermincola potens JR | firmicutes | 4C | C | G | C | C | G | C | E | H |
| 747365 | Thermodesulfobium narugense DSM 14796 | firmicutes | 4C | C | G | C | C | G | C | E | H |
| 349741 | Akkermansia muciniphila ATCC BAA-835 | verrucomic. | 4C | C | G | C | C | G | C | E | H |

**Table S1: Amino acid residues at key positions in the small (α) and large (β) subunits of the NiFe MBH known to influence O_2_ tolerance**

The table shows organisms utilised in the current study with their taxa ID and phylum. The NiFe MBH are classed as either O_2_ tolerant (6C) or standard (4C). The subsequent columns show six key positions in the small subunit, α60, 62, 63, 158,163, 192 and two in the large subunit, β73, 229 and their corresponding residues in the 177 organisms. The six positions in the small subunit coordinate the Fe-S cluster. The two positions in the large subunit interact with the Fe-S cluster. The sequences were acquired and classified as follows: The National Centre for Biotechnology Information (http://www.ncbi.nlm.nih.gov/) database of completed microbial genomes from isolated organisms, was mined using PSI-BLAST [1] and the amino acid sequence for the small subunit of the MBH from the well-characterised *R. eutropha* H16 (GI: 38637669 and *D. fructosovorans JJ* (GI: 205831249) as a query. We then acquired the corresponding sequences for the large subunit of these enzymes. Amino acid sequence from a total of 177 were utilised in all analyses. Following an alignment (Clustal Omega) of the small subunit, we observed a complete dichotomy between glycine and cysteine both at position 62 [Mx_58_CT**G**/**C**C] (*R. eutropha H16,* GI: 38637669 is used as a reference here and throughout) and position 163 [Mx_159_WG**G**/**C**VQ]. The four additional cysteines known to co-ordinate the proximal cluster were fixed in all 177 sequences with the exception of the cysteine in position 63 that occasionally changed to a proline (1/177), aspartic acid (8/177), or asparagine (10/177) residue. Given the multiple lines of evidence implicating the cysteine residues at position 62 and 163 in conveying O_2_ tolerance, following previous work, we defined tolerant enzymes as ones containing these substitutions, giving a total of six cysteines (6C group = TOL). The standard hydrogenases (SH) were those that had the glycine residues at these two positions (4C group = SH). In an alignment of sequence for the large subunit, at position 73, 102/114 4C enzymes had a glutamine (Q) residue, with the exception of 1/114 with a glycine (G) residue, 1/114 with a serine (S) residue, 3/114 with a glutamic acid (E) residue, and 7/114 with a Methionine (M) residue. For the 6C enzymes, 47/63 had the E residue with the exception of 2/63 with a G residue, and 14/63 with a Q residue. At position 229, 97% of organisms have the histidine (H) residue while five organisms (3 δ-proteobac. 2 unclassified bacteria) had an asparagine (N) residue instead.

| Small subunit | | |  |  |  |  |
| --- | --- | --- | --- | --- | --- | --- |
| ID | Length | Closet hit: Organism | GI | %Identities | Score | Expec |
| **20** | 362 | Rhodocyclaceae | 518758527 | 100.0 | 759.0 | 0 |
| 174 | 297 | Arcobacter butzleri RM4018 | 157737678 | 77.4 | 494.0 | 8 X 10^-174^ |
| **218** | 235 | Dechloromonas aromatica RCB | 71909599 | 96.6 | 456.0 | 1 X 10-^158^ |
| 100 | 235 | Sulfuricella denitrificans skB26 | 543962600 | 82.1 | 382.0 | 6 X 10^-129^ |
| 235 | 181 | Spirosoma linguale DSM 74 | 284038746 | 77.9 | 310.0 | 1 X 10^-102^ |
| 55 | 163 | Arcobacter butzleri RM4018 | 157737676 | 95.7 | 335.0 | 1 X 10^-111^ |
| 220 | 161 | Magnetospiril. magneticum AMB-1 | 83310214 | 75.8 | 270.0 | 2 X 10^-86^ |
| **230** | 151 | Azospirillum sp | 288961726 | 85.4 | 281.0 | 2 X 10^-91^ |
| 42 | 116 | Arcobacter nitrofigilis DSM 7299 | 296272894 | 84.5 | 212.0 | 1 X 10^-64^ |
| 151 | 115 | Geobacter sp | 322417996 | 81.7 | 192.0 | 4 X 10^-57^ |
| 155 | 110 | Sulfuricurvum kujiense DSM 16994 | 313683179 | 98.2 | 258.0 | 3 X 10^-79^ |
| 180 | 109 | Wolinella succinogenes DSM 1740 | 1346498 | 58.7 | 139.0 | 4 X 10^-37^ |
| 146 | 98 | Arcobacter sp | 384172724 | 88.8 | 188.0 | 1 X 10^-55^ |
| 14 | 98 | Geobacter metallireducens GS-15 | 404498180 | 71.4 | 160.0 | 8 X 10^-45^ |
| **229** | 92 | Methylocystis parvus | 515486359 | 92.4 | 161.0 | 5 X 10^-46^ |
| **133** | 55 | Mucilaginibacter paludis | 495785872 | 58.2 | 79.0 | 3 X 10^-15^ |
| 148 | 46 | Thiocystis violascens DSM 198 | 390950983 | 58.7 | 72.4 | 1 X 10^-13^ |
| 166 | 38 | Desulfitobacterium hafniense | 492328757 | 63.2 | 60.8 | 2 X 10^-9^ |
|  |  |  |  |  |  |  |
| Large Subunit | | |  |  |  |  |
| ID | Length | Closest hit: Organism | GI | %Identities | Score | Expec |
| **232** | 589 | Methyloversatilis universalis | 495338745 | 97.3 | 1204.0 | 0 |
| 54 | 580 | Arcobacter sp. L | 384172723 | 94.7 | 1158.0 | 0 |
| 139 | 304 | Dechloromonas aromatica RCB | 71909581 | 59.5 | 358.0 | 1 X 10^-116^ |
| 73 | 300 | Methanosarcina barkeri str Fusaro | 73669346 | 90.3 | 573.0 | 0 |
| 93 | 275 | Sulfuricurvum kujiense DSM 16994 | 313683178 | 90.9 | 534.0 | 0 |
| 158 | 220 | Thioalkalivib. thiocyanoxidans ARh 4 | 570728717 | 37.7 | 112.0 | 7 X 10^-26^ |
| 59 | 175 | delta proteobacterium NaphS2 | 493464592 | 73.1 | 275.0 | 6 X 10^-89^ |
| **6** | 156 | Methylocystis parvus | 515486358 | 89.1 | 305.0 | 5 X 10^-98^ |
| 183 | 148 | Desulfobac. autotrophicum HRM2 | 224369334 | 57.4 | 199.0 | 7 X 10^-58^ |
| 34 | 132 | Geobacter sulfurreducens PCA | 39995891 | 87.1 | 247.0 | 2 X 10^-76^ |
| 118 | 112 | Thermovibrio ammonificans HB-1 | 319790096 | 51.8 | 113.0 | 7 X 10^-27^ |
| 221 | 100 | delta proteobacterium MLMS-1 | 494505676 | 61.0 | 132.0 | 3 X 10^-34^ |
| 29 | 94 | Geobacter sp. M18 | 322417637 | 86.2 | 171.0 | 2 X 10^-50^ |
| 22 | 94 | uncultured bacterium | 406896436 | 66.0 | 137.0 | 1 X 10^-39^ |
| 231 | 81 | Sideroxydans lithotrophicus ES-1 | 291614080 | 79.0 | 137.0 | 1 X 10^-35^ |
| 215 | 77 | Thioalkalivib. thiocyanoxidans ARh 4 | 570728717 | 55.8 | 90.9 | 4 X 10^-20^ |
| 233 | 74 | Clostridium cellulolyticum H10 | 220928501 | 59.5 | 104.0 | 3 X 10^-24^ |
| **39** | 69 | Dechloromonas aromatica RCB | 71909598 | 95.7 | 144.0 | 7 X 10^-39^ |
| 145 | 68 | uncultured bacterium | 406888611 | 63.2 | 104.0 | 3 X 10^-27^ |
| 99 | 67 | Dechloromonas aromatica RCB | 71909598 | 79.1 | 122.0 | 8 X 10^-31^ |
| 35 | 67 | Thiobacillus denitrificans | 516742072 | 79.1 | 124.0 | 9 X 10^-32^ |
| 122 | 60 | Dehalococcoides mccartyi VS | 270307563 | 63.3 | 95.9 | 1 X 10^-21^ |
| 5 | 60 | S. enterica serov. Enteritidis. | 604196331 | 60.0 | 85.9 | 2 X 10^-19^ |
| 161 | 47 | Deferribacter desulfuricans SSM1 | 291278509 | 74.5 | 91.7 | 4 X 10^-20^ |
| 206 | 45 | Desulfovibrio longus | 550907540 | 77.8 | 85.1 | 5 X 10^-18^ |
| **181** | 38 | Asticcacaulis sp. AC466 | 557832447 | 100.0 | 86.7 | 2 X 10^-18^ |

**Table S2: BLAST hits from the non-redundant sequence database for *de novo* metagenomic fragment queries**

The data are sorted by fragment size (column labelled ‘length’), with full-length protein sequences appearing first. Sequences that segregated with known 6C enzymes on the phylogenetic trees for both the small (Figure S1, 2, S4) and large (Figure S3, S5) subunits appear in boldface.


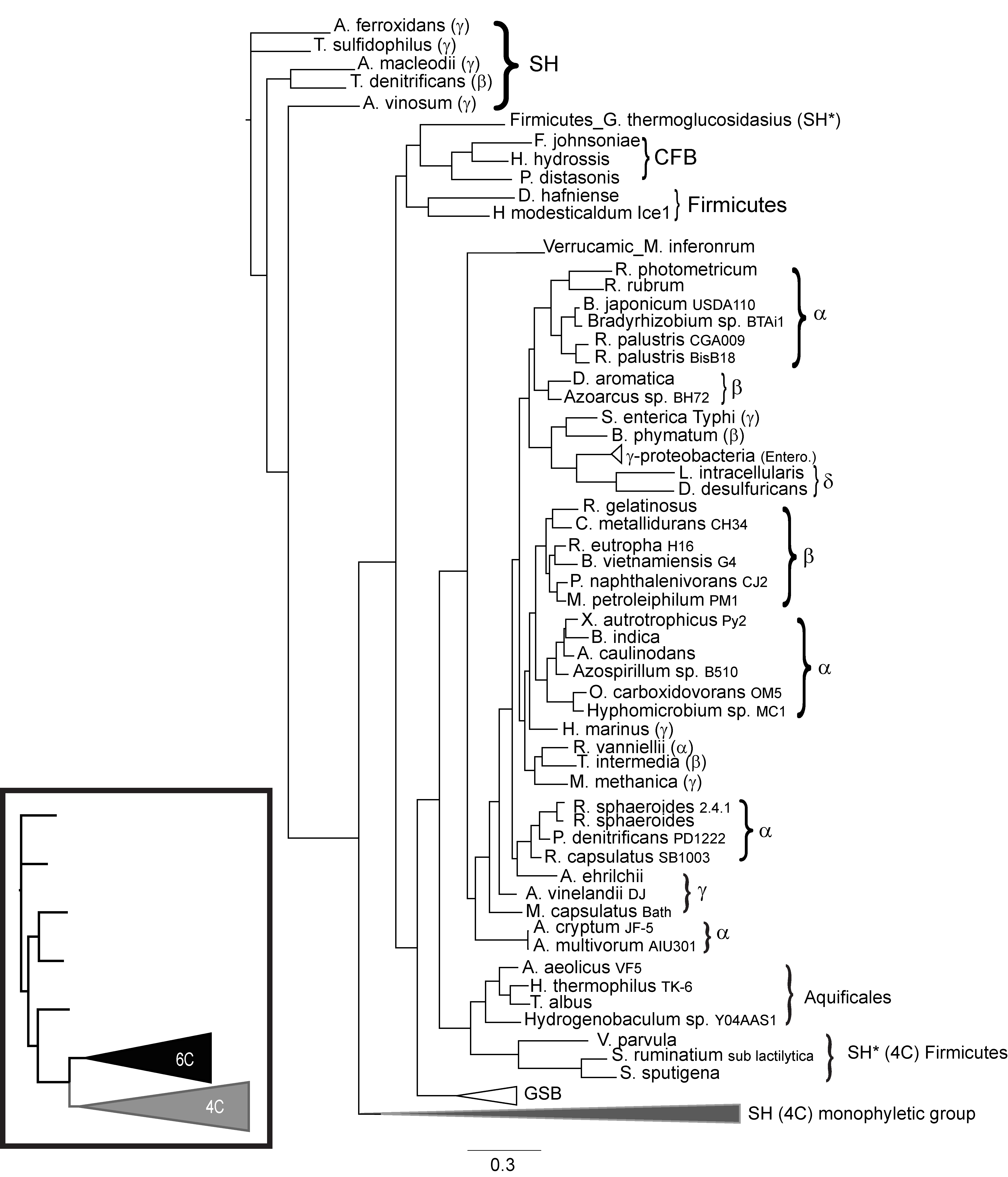


**Figure S1: Phylogenetic tree of sequences for the enzyme’s small subunit-6C sequences**

A phylogenetic tree of group 1 NiFe MBH small subunit sequences ascertained from the NCBI public database. The complete structure is pictured inset, with the expanded portion of 6C enzymes shown in black. The 4C group (grey triangle) is also known as “SH” for standard hydrogenase. It has been collapsed, but can be viewed in detail in Figure S2. This is a consensus tree constructed in MrBayes v3.2 with a potential scale reduction factor = 1.000. The scale refers to 0.3 expected changes per site. Unless indicated by “SH”, all enzymes are 6C. Collapsed groups are shown as white triangles with a black outline. The ‘oldest’ or least derived hydrogenases on the tree are all sulphur oxidizing γ- proteobacteria, with the exception of *Thiobacillis denitrificans*, a sulphur reducing β-proteobacteria. All are standard hydrogenases (SH/4C) enzymes. Following the split of *Allochromatium vinosum* from this pool of early ancestors, the tree indicates the crucial event (posterior probability = 1.000) that produced two monophyletic groups, the tolerant (6C; this figure) and the standard (4C; Figure S2) hydrogenases. This change appears to divide these two groups over evolutionary time with the exception of four standard (4C/SH*) enzymes from the Firmicutes phylum that appear within the cluster of tolerant (6C) hydrogenases. These are *Geobacillus thermoglucosidasius C56-YS93* (GI: 336235542) that clusters with the 6C Firmicutes and the CFB (Chlorobi Flavobacteriales Bacterioidetes) group, and three additional species, *Selenomonas ruminantium* (GI: 383753115), *Selonomonas sputigena* (GI: 260887607), and *Veillonella parvula* (GI: 282850486) that share a common ancestor with the 6C *Aquificales*. The tree topology presented here is different from the study of Pandelia et al (2012), although the conclusions are very similar. We utilized a Bayesian inference with 2.5 million simulations and 177 sequences, which is more statistically robust in comparison to the neighbour-joining method employed by Pandelia et al. (2012), which is most likely the reason for the different topologies.


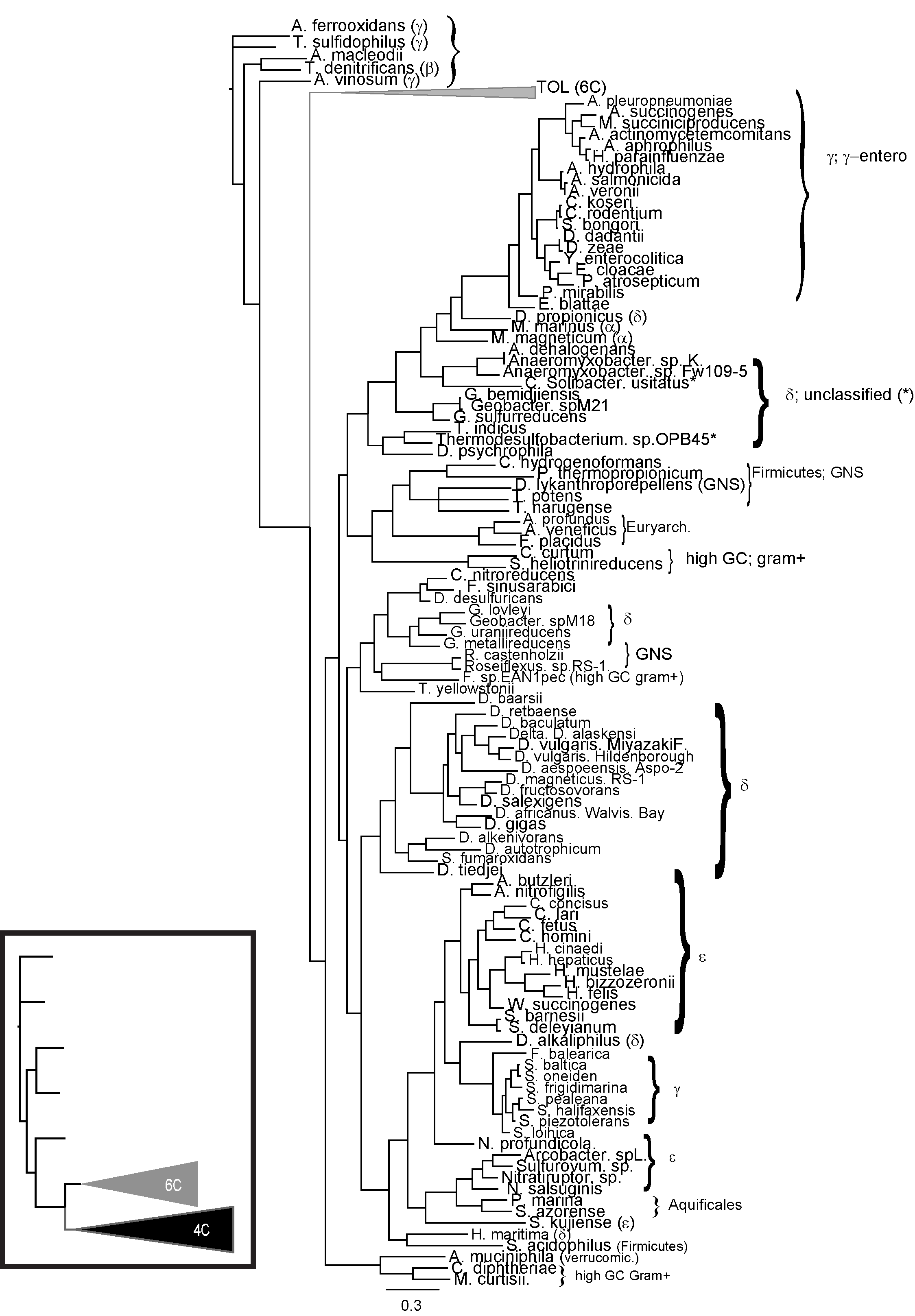


**Figure S2: Phylogenetic tree of sequences for the enzyme’s small subunit-4C sequences**

A phylogenetic tree of NiFe MBH hydrogenase small subunit sequences ascertained from the NCBI public database. The complete structure is pictured inset, with the expanded portion of 4C enzymes shown in black. The 6C group (grey triangle) has been collapsed, but can be viewed in detail in Figure S1. This is a consensus tree constructed in MrBayes v3.2 with a potential scale reduction factor = 1.000. The scale refers to 0.3 expected changes per site. The four “ancestral” enzymes are all standard hydrogenase (SH/4C) enzymes. An asterisks mark species that have not been classified into a specific phylum group. Unless otherwise indicated, all enzymes are standard hydrogenases, indicated by 4C.


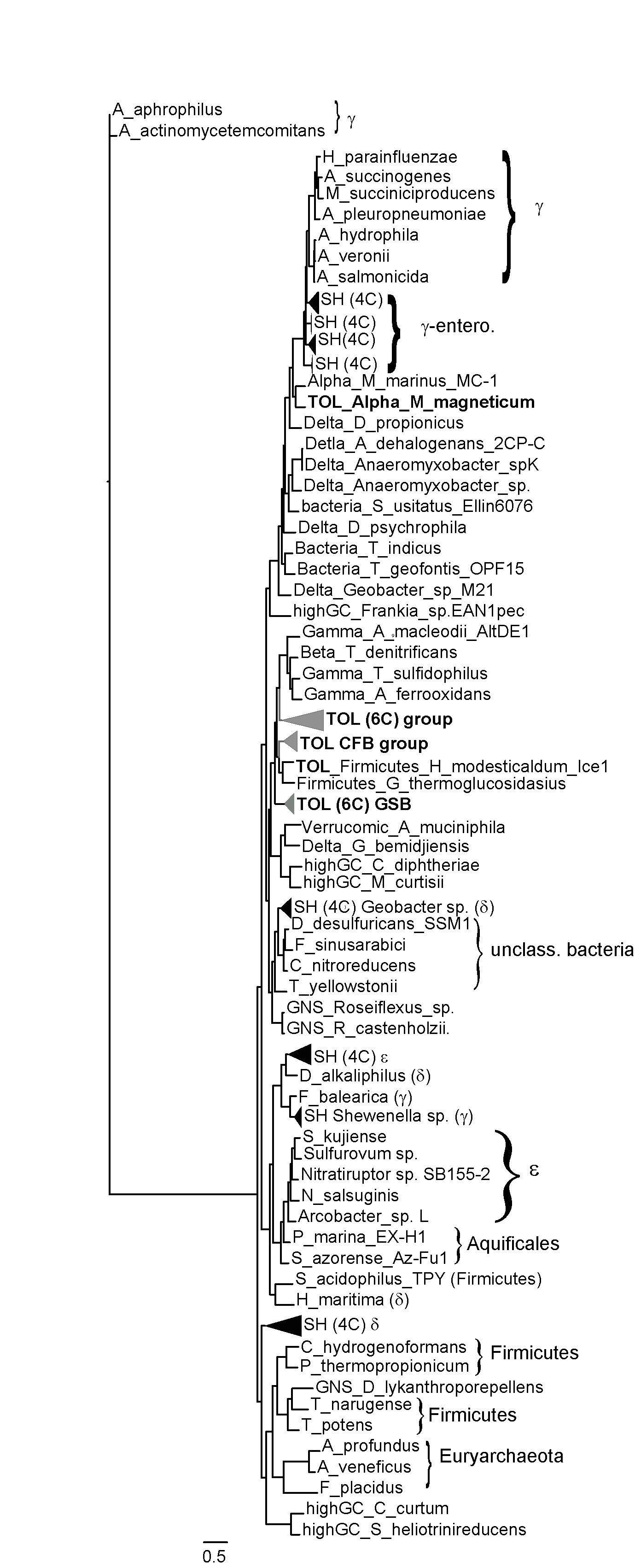


**Figure S3: Phylogenetic tree of sequences for the enzyme’s large subunit**

A phylogenetic tree of group 1 NiFe MBH large subunit sequences ascertained from a public database. The 6C/TOL groups have been collapsed and are represented by grey triangles. The black triangles also represent collapsed groups. The name for each of these groups appears beside the triangle. This is a consensus tree constructed in MrBayes v3.2 with a potential scale reduction factor = 1.000. The scale refers to 0.5 expected changes per site.


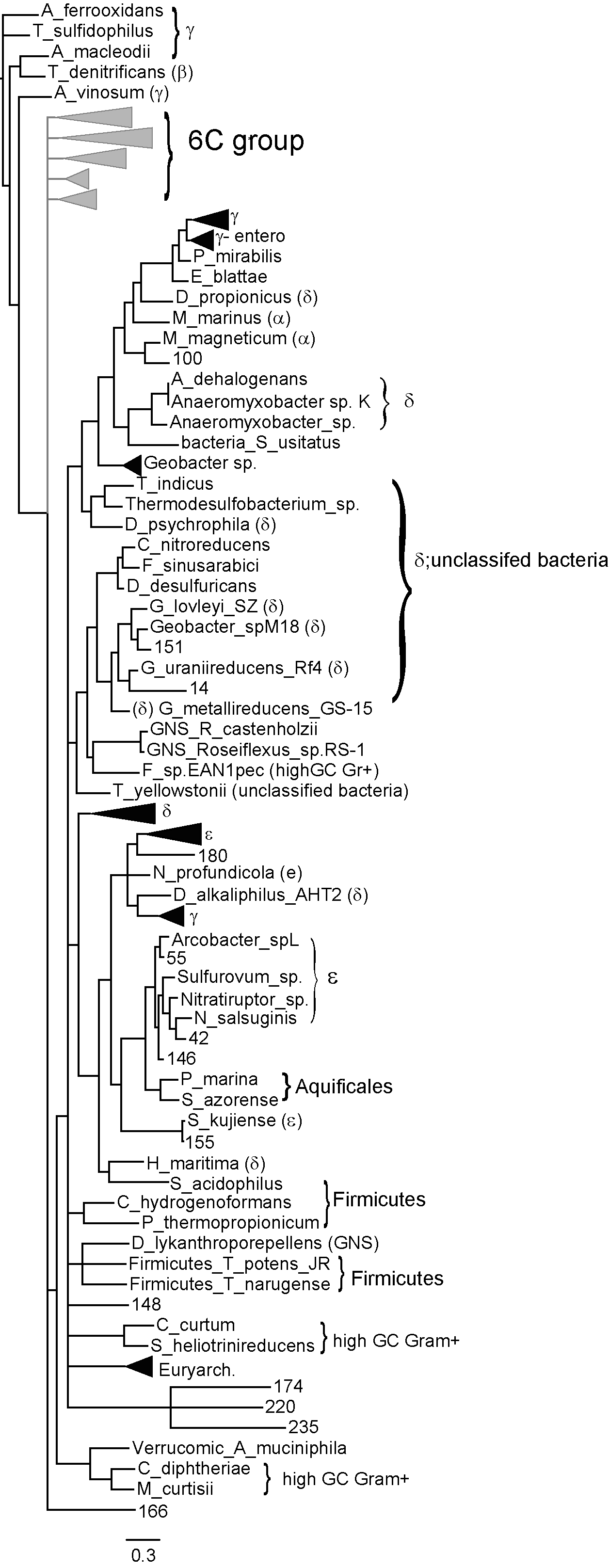


**Figure S4: Tree of sequences for the enzyme’s small subunit with**

**segregating *de novo* environmental metagenomic fragments-4C**

**sequences**

The phylogenetic tree of the small subunit of the NiFe MBH from the public databases reconstructed to include *de novo* sequence fragments recovered from a sample environmental metagenome. This figure provides the detailed topology of the 4C enzymes. The 6C group (grey triangles) has been collapsed, but can be viewed in detail in Figure 4. On this tree, the fragments 100, 151, 14, 180, 55, 42, 146, and 155 segregate close to known 4C enzymes, while fragments 148, 174, 220, and 235 appear to be distinct groups. The scale refers to 0.3 expected changes per site.


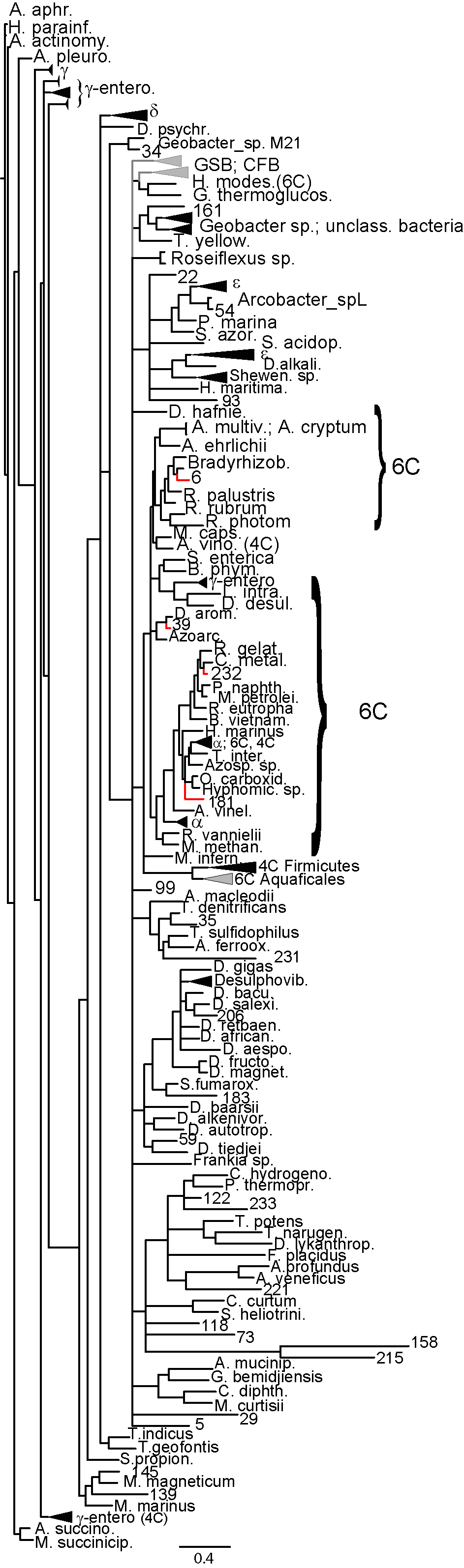


**Figure S5:** **Tree of sequences for the enzyme’s large subunit with segregating *de novo* environmental metagenomic fragments**

A phylogenetic tree of sequences of the large subunit of the NiFe MBH from the NCBI database reconstructed to include *de novo* sequence fragments recovered from a sample environmental metagenome. For clarity, phylum names have been omitted, but can be found in Table S1 [Table S1: Amino acid residues at key positions in the small (α) and large (β) subunits of the NiFe MBH known to influence O_2_ tolerance]. A number of groups have been collapsed and are represented by either grey (6C) or black triangles. The scale refers to 0.4 expected changes per site. Fragment numbers 6, 39, 232, and 181 segregating with the 6C enzymes and are connected by red lines indicate branches supported by very low probabilities. Of these, sequence 232 is nearly full-length and segregates with *R. gelatinosus* and *C. metallidurans*, two well-characterised O_2_ tolerant hydrogenases, suggesting that it too is an O_2_ tolerant hydrogenase from the β-proteobacteria phylum.

References:

1. Altschul SF, Madden TL, Schaffer AA, Zhang J, Zhang Z, Miller W, Lipman DJ (1997) Gapped BLAST and PSI-BLAST: a new generation of protein database search programs. Nucleic acids research 25 (17):3389-3402

2. Vignais PM, Billoud B, Meyer J (2001) Classification and phylogeny of hydrogenases. FEMS microbiology reviews 25 (4):455-501

3. Whelan S, Goldman N (2001) A general empirical model of protein evolution derived from multiple protein families using a maximum-likelihood approach. Molecular biology and evolution 18 (5):691-699

4. Peng Y, Leung HC, Yiu SM, Chin FY (2012) IDBA-UD: a de novo assembler for single-cell and metagenomic sequencing data with highly uneven depth. Bioinformatics 28 (11):1420-1428. doi:10.1093/bioinformatics/bts174

5. Seemann T (2014) Prokka: rapid prokaryotic genome annotation. Bioinformatics 30 (14):2068-2069. doi:10.1093/bioinformatics/btu153
